# SWATH-MS proteome profiling data comparison of DJ-1, Parkin, and PINK1 knockout rat striatal mitochondria

**DOI:** 10.1016/j.dib.2016.09.031

**Published:** 2016-09-23

**Authors:** Kelly L. Stauch, Lance M. Villeneuve, Phillip R. Purnell, Sanjit Pandey, Chittibabu Guda, Howard S. Fox

**Affiliations:** a Department of Pharmacology and Experimental Neuroscience, University of Nebraska Medical Center, Omaha, NE 68198, United States; b Department of Genetics, Cell Biology and Anatomy, University of Nebraska Medical Center, Omaha, NE 68198, United States; c Bioinformatics and Systems Biology Core, University of Nebraska Medical Center, Omaha, NE, United States

**Keywords:** Mitochondria, Parkinson׳s disease, SWATH-MS

## Abstract

This article reports changes in the striatal non-synaptic mitochondrial proteome of DJ-1, Parkin, and PINK1 knockout (KO) rats at 3 months of age. DJ-1, Parkin, and PINK1 mutations cause autosomal-recessive parkinsonism. It is thought that loss of function of these proteins contributes to the onset and pathogenesis of Parkinson׳s disease (PD). As DJ-1, Parkin, and PINK1 have functions in the regulation of mitochondria, the dataset generated here highlights protein expression changes, which can be helpful for understanding pathological mitochondrial alterations. In total, 1281 proteins were quantified and 25, 37, and 15 proteins were found to exhibit differential expression due to DJ-1, Parkin, and PINK1 deficiency, respectively. All quantification can be found in the supplemental table and can be searched online at http://genome.unmc.edu/mitorat/index.html. Further, mitochondrial respiration was measured to evaluate mitochondrial function in the striatum of DJ-1, Parkin, and PINK1 KO rats, which was significantly changed only in the DJ-1 KOs.

**Specifications table**

**Table:** 

Subject area	*Biology*
More specific subject area	*Neurobiology*
Type of data	*Table*, *Graph*
How data was acquired	*SWATH-MS*: *TripleTOF 5600* (*SCIEX*), *Mitochondrial respiration*: *Seahorse XF24 Extracellular Flux Analyzer*
Data format	*Analyzed*
Experimental factors	*Genetic ablation of Park7* (*DJ-1*), *Park2* (*Parkin*), *or Park6* (*PINK1*) (*Parkinson׳s disease animal model*)
Experimental features	*Mitochondria were isolated from rat striatum*, *SWATH-MS of mitochondrial peptides*
Data source location	*Omaha*, *NE*
Data accessibility	*The data are included in this article*

**Value of the data**

• The data describe techniques to isolate intact functional rat striatal non-synaptic mitochondria for proteome and respiratory profiling experiments.
• The data can be used as a reference regarding the common and distinct striatal non-synaptic mitochondrial proteome alterations associated with DJ-1, Parkin, and PINK1 deficiency.
• As loss of function of any of these three proteins is involved in PD [1], changes in the proteome and respiratory function of striatal non-synaptic mitochondria in response to DJ-1, Parkin, and PINK1 deficiency may be of wide interest to researchers investigating therapeutic targets and mechanisms of disease progression.

## 1 Data

**Fig. 1:**
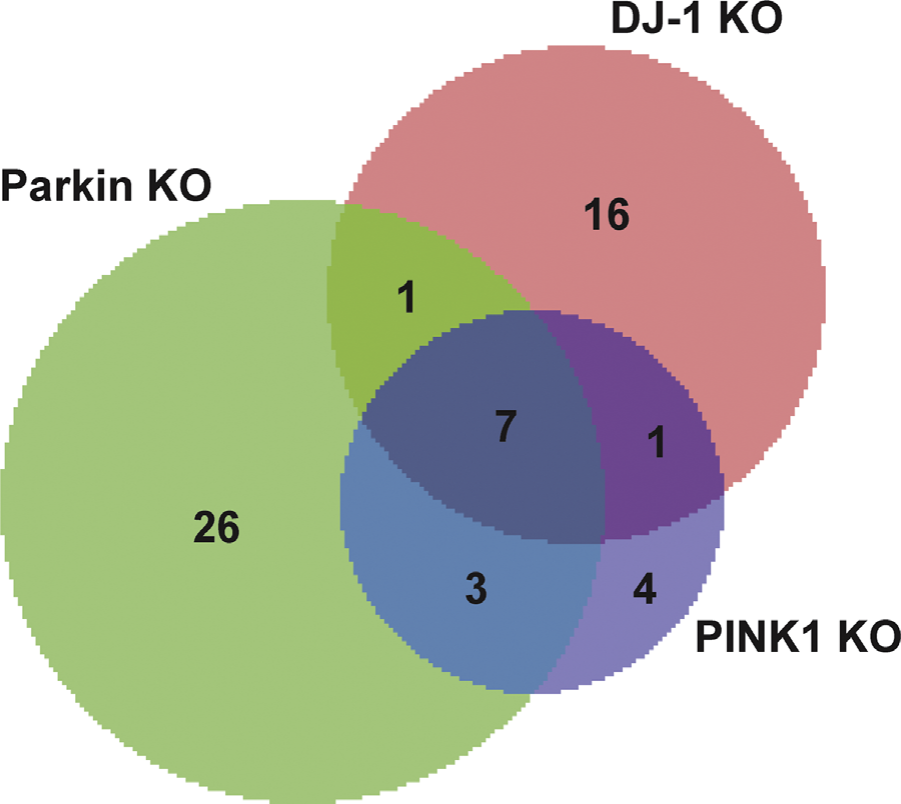
Venn diagram of the common and distinct differentially expressed striatal mitochondrial proteins from DJ-1, Parkin, and PINK1 KO rats when compared to control rats identified by SWATH-MS.

**Fig. 2:**
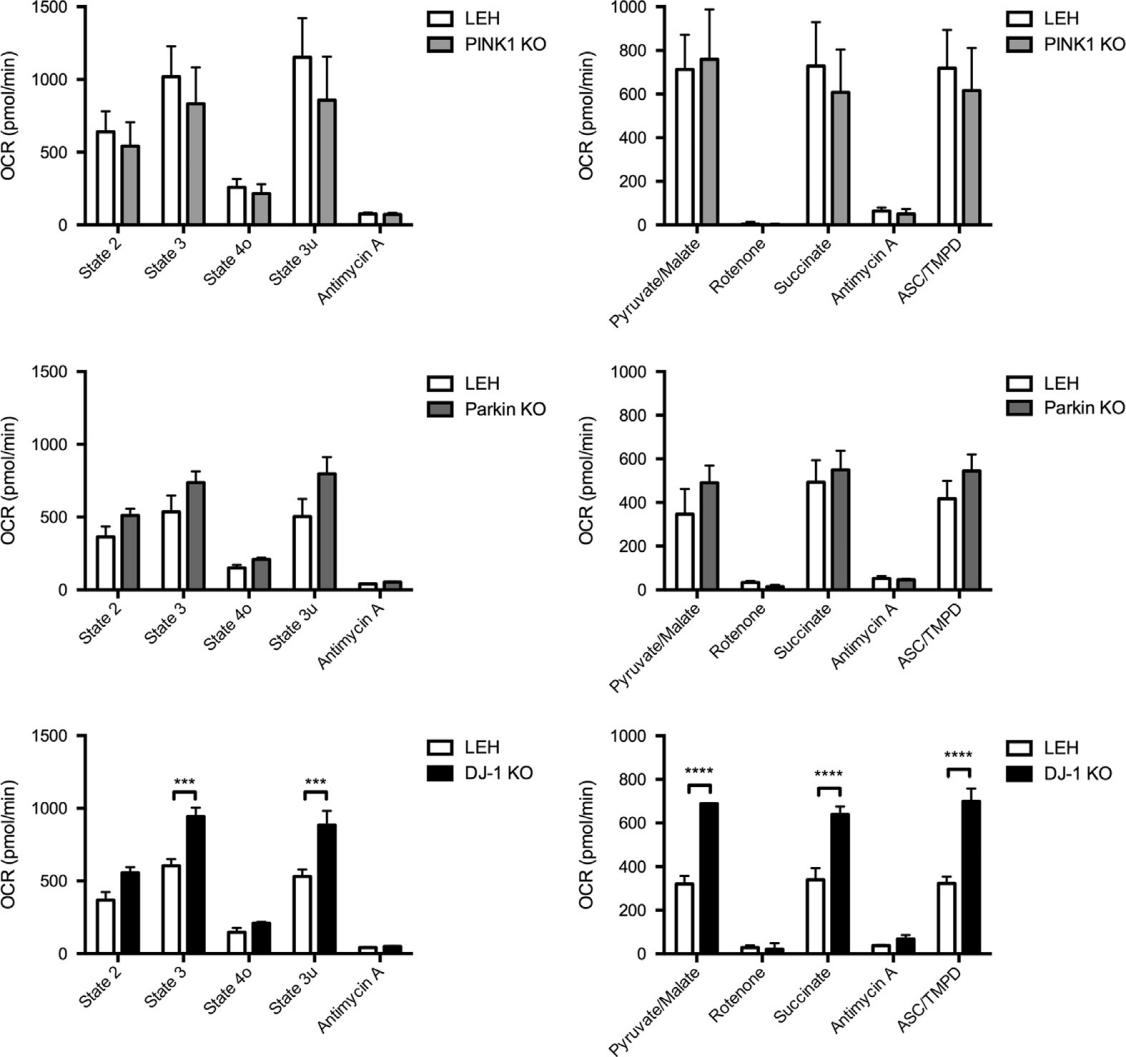
Effect of loss of DJ-1, Parkin, and PINK1 on striatal non-synaptic mitochondrial respiration. For the coupling assay oxygen consumption rate (OCR) was determined in the presence of succinate (State 2, basal respiration) followed by subsequent injections of ADP (State 3, phosphorylating respiration), oligomycin (State 4o, non-phosphorylating respiration), FCCP (State 3u, uncoupled respiration), and antimycin A (complex III inhibitor). For the electron flow assay OCR was measured in the presence of pyruvate/malate (complex I driven respiration) followed by subsequent injections of rotenone (complex I inhibitor), succinate (complex II driven respiration), antimycin A (complex III inhibitor), and ascorbate (ASC)/TMPD (complex IV driven respiration). Values are the means±SEM; ***p<0.001 vs. LEH; ****p<0.0001 vs. LEH; Two-way ANOVA with Sidak׳s post hoc test.

The data presented here show the comparative SWATH-MS analysis of the striatal non-synaptic mitochondrial proteome of 3-month-old control and knockout (DJ-1, Parkin, and PINK1) rats (Supplementary Table 1, Fig. 1, and Table 1), as well as mitochondrial respiration (Fig. 2) as an indicator of mitochondrial function.

## 2 Experimental design, materials and methods

### 2.1 Animals

Male knockout (DJ-1, Parkin, and PINK1) and the Long Evans Hooded (LEH) control rats [2] at age 3 months were used for the experiments, which were conducted within National Institute of Health approved guidelines with the approval of the University of Nebraska Medical Center Institutional Animal Care and Use Committee.

### 2.2 Mitochondrial isolation

Each rat was anesthetized and sacrificed, the brain was removed, and striatal tissue was quickly dissected. Non-synaptic mitochondria were isolated via Percoll gradient centrifugation as previously described [3]. The non-synaptic mitochondrial pellets were resuspended in a minimal volume of mitochondrial assay solution (MAS, 1×): 70 mM sucrose, 220 mM mannitol, 10 mM KH_2_PO_4_, 5 mM MgCl_2_, 2 mM HEPES, and 1 mM EGTA (pH 7.2) and the Pierce BCA Protein Assay Kit with Albumin Standards (Thermo Fischer Scientific) was used for protein quantification.

### 2.3 Preparation of mitochondrial peptides for SWATH-MS

The non-synaptic mitochondria used for mass spectrometry were lysed in 4% sodium dodecyl sulfate and the Pierce 660 nm Protein Assay with Albumin Standards (Thermo Fisher Scientific) was used for protein quantification. The non-synaptic mitochondrial peptides were prepared for mass spectrometry following the filter aided sample preparation method [4] as described previously [5], [6], [7], [8]. The non-synaptic mitochondrial peptides were quantified using the Scopes method [9] on a NanoDrop 2000 UV–vis Spectrophotometer (Thermo Fisher Scientific).

### 2.4 SWATH data acquisition

The striatal non-synaptic mitochondrial peptides for each independent biological replicate (*n*=4 for each strain) were analyzed by SWATH-MS data-independent acquisition (DIA) mode on a TripleTOF 5600 (SCIEX) followed by targeted data extraction as described previously [5], [6], [7], [8]. For peptide identification, our published reference spectral library was used [5], [6], [7], [8], which contains 3241 proteins identified with high confidence (greater than 99%) that passed the global false discovery rate (FDR) from fit analysis using a critical FDR of 1%. The Cyber-T Web server (http://cybert.ics.uci.edu/) [10] was used to assess significance via a *t*-test using a Bayesian regularization method for quantitative mass spectrometry. Proteins deemed as differentially expressed were *p*-values<0.05 from the pairwise post-hoc test (TukeyHSD) that also exhibited Posterior Probability of Differential Expression (PPDE)-values>0.95. BioVenn [11] was used to visualize the lists of differentially expressed proteins for each KO strain when compared to the control strain as a Venn diagram (Fig. 1).

### 2.5 Mitochondrial respiration

Mitochondrial respiration was completed using a Seahorse XF24 Extracellular Flux Analyzer (Seahorse Bioscience) and oxygen consumption rates (OCR) were measured using 3–4 technical replicate wells for each independent biological replicate (*n*=3 for each strain). The coupling and electron flow assays were completed as previously described [3], [5], [12] using 7.5 μg of striatal non-synaptic mitochondria per well. The Seahorse Wave 2.2.0 software package was used for data calculation and graphs (Fig. 2) were generated in Prism (GraphPad Software). Statistical analyses were conducted in Prism using ANOVA and Sidak׳s multiple comparisons post-hoc testing on the complete Seahorse assay data set.

## Figures and Tables

**Table 1:** List of differentially expressed striatal mitochondrial proteins in all three KO rat strains (DJ-1, Parkin, and PINK1) compared to the control rat strain. Protein expression values listed are log_2_ (KO/LEH).

UniProt	Protein	Gene	DJ-1 KO/LEH	*p*-value	Parkin KO/LEH	*p*-Value	PINK1 KO/LEH	*p*-Value
Q4QQV4	Dead end homolog 1	Hars	−1.70	0.002	−2.63	0.000	−3.30	0.000
M0R5E7	Protein Ttc37	Ttc37	−2.72	0.000	−3.60	0.000	−3.00	0.000
P80432	Cytochrome c oxidase subunit 7C	Cox7c	−2.08	0.002	−2.54	0.001	−1.55	0.009
F1M9C9	Protein Hars2	Hars2	−1.60	0.009	−3.23	0.000	−1.54	0.012
P21588	5′nucleotidase	Nt5e	−1.78	0.006	−2.34	0.002	−1.51	0.014
Q5XI22	Acetyl-CoA acetyltransferase	Acat2	−1.64	0.040	−2.57	0.008	−2.97	0.005
O35889	Afadin	Mllt4	−2.32	0.017	−3.10	0.007	−2.33	0.017

## Appendix A Supplementary material

**Supplementary Table 1** SWATH-MS proteomic dataset of 3-month-old DJ-1, Parkin, and PINK1 rat striatal non-synaptic mitochondria.
